# Supplementary material for: Prognostic Value of SDF-1α Expression in Patients with Esophageal Squamous Cell Carcinoma Receiving Esophagectomy
Source: Cancers (Basel). 2020 Apr 25;12(5):1067. doi: 10.3390/cancers12051067 (PMC7281421; doi:10.3390/cancers12051067)
Supplement: Supplementary file 1 [file cancers-12-01067-s001.pdf]

Supplementary Materials

Prognostic Value of SDF-1α Expression in Patients with Esophageal Squamous Cell Carcinoma Receiving Esophagectomy

Yen-Hao Chen, Shau-Hsuan Li, Hung-I Lu and Chien-Ming Lo

Table S1. The IHC reading of SDF-1α.

Patient List	Pathologist 1			Pathologist 2			Final	
	SDF-1α Positive	SDF-1α Intensity	SDF-1α Score	SDF-1α Positive	SDF-1α Intensity	SDF-1α Score	Mean of SDF-1α Socre	SDF-1α Overexpression (0 = Low Expression; 1 = Overexpression)
1	2	2	4	2	2	4	4	0
2	2	2	4	2	2	4	4	0
3	1	1	1	1	1	1	1	0
4	1	1	1	1	1	1	1	0
5	1	1	1	1	1	1	1	0
6	1	2	2	1	2	2	2	0
7	2	1	2	2	1	2	2	0
8	2	1	2	2	2	4	3	0
9	0	0	0	0	0	0	0	0
10	0	0	0	0	0	0	0	0
11	2	2	4	2	2	4	4	0
12	1	2	2	1	2	2	2	0
13	2	1	2	2	1	2	2	0
14	1	1	1	1	1	1	1	0
15	2	1	2	2	1	2	2	0
16	1	1	1	1	1	1	1	0
17	2	2	4	2	2	4	4	0
18	3	1	3	3	1	3	3	0
19	2	2	4	2	1	2	3	0
20	3	1	3	3	1	3	3	0
21	2	2	4	2	2	4	4	0
22	3	1	3	3	1	3	3	0
23	0	0	0	0	0	0	0	0
24	1	1	1	1	1	1	1	0
25	1	2	2	1	2	2	2	0
26	2	2	4	2	2	4	4	0
27	3	1	3	3	1	3	3	0
28	2	1	2	2	1	2	2	0
29	1	1	1	1	1	1	1	0
30	4	1	4	4	1	4	4	0
31	1	1	1	1	1	1	1	0
32	3	1	3	3	1	3	3	0
33	2	1	2	2	1	2	2	0
34	3	2	6	3	2	6	6	1
35	1	2	2	1	2	2	2	0
36	4	2	8	3	2	6	7	1
37	2	1	2	2	2	4	3	0
38	0	0	0	0	0	0	0	0
39	4	1	4	4	1	4	4	0
40	2	2	4	2	2	4	4	0

41	4	2	8	4	2	8	8	1
42	3	1	3	3	1	3	3	0
43	3	2	6	4	2	8	7	1
44	0	0	0	0	0	0	0	0
45	1	2	2	1	2	2	2	0
46	3	2	6	3	2	6	6	1
47	3	1	3	3	1	3	3	0
48	2	2	4	2	2	4	4	0
49	2	2	4	2	2	4	4	0
50	0	0	0	0	0	0	0	0
51	4	1	4	4	2	8	6	1
52	0	0	0	0	0	0	0	0
53	3	2	6	3	2	6	6	1
54	3	2	6	3	2	6	6	1
55	4	2	8	4	2	8	8	1
56	3	2	6	3	2	6	6	1
57	3	2	6	3	2	6	6	1
58	3	2	6	3	2	6	6	1
59	3	2	6	3	2	6	6	1
60	3	2	6	3	2	6	6	1
61	3	2	6	3	2	6	6	1
62	3	2	6	3	2	6	6	1
63	4	2	8	4	2	8	8	1
64	3	2	6	3	2	6	6	1
65	3	2	6	3	2	6	6	1
66	3	2	6	3	2	6	6	1
67	3	2	6	3	2	6	6	1
68	3	2	6	3	2	6	6	1
69	4	2	8	4	2	8	8	1
70	3	2	6	3	2	6	6	1
71	3	2	6	3	2	6	6	1
72	3	2	6	3	2	6	6	1
73	4	2	8	4	2	8	8	1
74	3	2	6	3	2	6	6	1
75	4	2	8	4	2	8	8	1
76	3	2	6	3	2	6	6	1
77	3	2	6	3	2	6	6	1
78	2	1	2	2	2	4	3	0
79	2	1	2	2	1	2	2	0
80	2	2	4	2	2	4	4	0
81	0	0	0	0	0	0	0	0
82	2	1	2	2	1	2	2	0
83	0	0	0	0	0	0	0	0
84	2	2	4	2	2	4	4	0
85	1	1	1	1	1	1	1	0
86	3	1	3	3	1	3	3	0
87	0	0	0	0	0	0	0	0
88	2	2	4	2	2	4	4	0
89	0	0	0	0	0	0	0	0
90	2	2	4	2	2	4	4	0
91	1	2	2	1	2	2	2	0
92	1	2	2	1	2	2	2	0
93	1	2	2	1	2	2	2	0
94	3	1	3	3	1	3	3	0
95	2	1	2	2	1	2	2	0
96	2	2	4	2	2	4	4	0

97	0	0	0	0	0	0	0	0
98	1	1	1	1	1	1	1	0
99	1	2	2	1	2	2	2	0
100	0	0	0	0	0	0	0	0
101	2	2	4	2	2	4	4	0
102	2	1	2	2	1	2	2	0
103	2	1	2	2	1	2	2	0
104	2	2	4	2	2	4	4	0
105	2	1	2	2	1	2	2	0
106	1	1	1	1	1	1	1	0
107	1	2	2	1	2	2	2	0
108	2	2	4	2	2	4	4	0
109	1	1	1	1	1	1	1	0
110	1	2	2	1	2	2	2	0
111	3	1	3	3	1	3	3	0
112	2	0	0	2	0	0	0	0
113	1	2	2	1	2	2	2	0
114	1	2	2	1	2	2	2	0
115	0	0	0	0	0	0	0	0
116	2	1	2	2	1	2	2	0
117	1	1	1	1	1	1	1	0
118	3	2	6	3	2	6	6	1
119	4	2	8	4	2	8	8	1
120	3	2	6	3	2	6	6	1
121	1	1	1	1	1	1	1	0
122	3	1	3	3	1	3	3	0
123	1	2	2	1	2	2	2	0
124	4	1	4	4	2	8	6	1
125	2	2	4	2	2	4	4	0
126	4	2	8	4	2	8	8	1
127	3	2	6	3	2	6	6	1
128	3	2	6	3	2	6	6	1
129	4	2	8	4	2	8	8	1
130	4	2	8	4	2	8	8	1
131	4	2	8	4	2	8	8	1
132	3	2	6	3	2	6	6	1
133	4	2	8	4	2	8	8	1
134	3	2	6	3	2	6	6	1
135	3	2	6	3	2	6	6	1
136	2	2	6	2	2	6	6	1
137	4	2	8	4	2	8	8	1
138	3	2	6	3	2	6	6	1
139	4	2	8	4	2	8	8	1
140	4	2	8	4	2	8	8	1
141	3	2	6	4	2	8	7	1
142	3	1	3	3	1	3	3	0
143	2	2	4	2	2	4	4	0
144	2	1	2	2	1	2	2	0
145	4	2	8	4	2	8	8	1
146	1	1	1	1	1	1	1	0
147	2	1	2	2	1	2	2	0
148	1	2	2	1	2	2	2	0
149	2	2	4	2	2	4	4	0
150	0	0	0	0	0	0	0	0
151	2	1	2	2	1	2	2	0
152	2	1	2	2	1	2	2	0

153	0	0	0	0	0	0	0	0
154	2	2	4	2	2	4	4	0
155	2	2	4	2	2	4	4	0
156	2	1	2	2	1	2	2	0
157	3	1	3	3	1	3	3	0
15	2	1	2	2	1	2	2	0
159	3	2	6	3	2	6	6	1
160	2	2	4	2	2	4	4	0
161	4	2	8	4	2	8	8	1
162	0	0	0	0	0	0	0	0
163	0	0	0	0	0	0	0	0
164	3	2	6	3	2	6	6	1
165	4	2	8	4	2	8	8	1
166	3	2	6	3	2	6	6	1
167	4	2	8	4	2	8	8	1
168	3	2	6	3	2	6	6	1
169	3	2	6	3	2	6	6	1

© 2020 by the authors. Licensee MDPI, Basel, Switzerland. This article is an open access article distributed under the terms and conditions of the Creative Commons Attribution (CC BY) license (http://creativecommons.org/licenses/by/4.0/).
